# High expression of PDGFR-β in prostate cancer stroma is independently associated with clinical and biochemical prostate cancer recurrence

**DOI:** 10.1038/srep43378

**Published:** 2017-02-24

**Authors:** Yngve Nordby, Elin Richardsen, Mehrdad Rakaee, Nora Ness, Tom Donnem, Hiten R. H. Patel, Lill-Tove Busund, Roy M. Bremnes, Sigve Andersen

**Affiliations:** 1 Dept Clinical Medicine, The Arctic University of Norway, Tromso, Norway; 2 Dept Urology, University Hospital of North Norway, Tromso, Norway; 3 Dept Clinical Pathology, University Hospital of North Norway, Tromso, Norway; 4 Dept Medical Biology, The Arctic University of Norway, Tromso, Norway; 5 Dept Oncology, University Hospital of North Norway, Tromso, Norway

## Abstract

Due to a lack of sufficient diagnostic tools to predict aggressive disease, there is a significant overtreatment of patients with prostate cancer. Platelet derived growth factors (PDGFs) and their receptors (PDGFRs) are key regulators of mesenchymal cells in the tumor microenvironment, and has been associated with unfavorable outcome in several other cancers. Herein, we aimed to investigate the prognostic impact of PDGFR-β and its ligands (PDGF-B and PDGF-D) in a multicenter prostatectomy cohort of 535 Norwegian patients. Using tissue microarrays and immunohistochemistry, the expression of ligands PDGF-B and PDGF-D and their corresponding receptor, PDGFR-β, was assessed in neoplastic tissue and tumor-associated stroma. PDGFR-β was expressed in benign and tumor associated stroma, but not in epithelium. High stromal expression of PDGFR-β was independently associated with clinical relapse (HR = 2.17, p = 0.010) and biochemical failure (HR = 1.58, p = 0.002). This large study highlights the prognostic importance of PDGFR-β expression, implicating its involvement in prostate cancer progression even in early stage disease. Hence, analyses of PDGFR-β may help distinguish which patients will benefit from radical treatment, and since PDGFR-β is associated with relapse and shorter survival, it mandates a focus as a therapeutic target.

Prostate cancer (PC) is the most frequent malignancy in men^1^. Despite a relatively low mortality rate, the sheer PC incidence rate makes it the second most common cause of male cancer death in developed countries. Differentiation between patients with an aggressive and potentially deadly form of PC versus patients with indolent disease remains a challenge. Contemporary risk stratification leads to a significant overtreatment (radical therapy), but possibly also an undertreatment of some patients^2^,^3^,^4^. There is a definite need for better prognostic tools to aid in the prediction of which patients will benefit from curative treatment.

The platelet derived growth factor ligands (PDGFs) and their receptors (PDGFRs) have emerged as key regulators of cell growth and division, and mediate significant impact on malignant cells and the tumor microenvironment^5^. As potent mitogens for cells of mesenchymal origin, the PDGFs are important regulatory proteins for fibroblasts, smooth muscle cells and glial cells. They are involved in embryonic development, cell proliferation, cell migration and stimulate wound healing in the adult^6^. In particular, these factors play a significant role in angiogenesis in which mutational activation or upregulation of the PDGFs or PDGFRs may lead to uncontrolled blood vessel formation and cancer.

There are five different known isoforms of PDGF ligands: PDGF-AA (PDGF-A), PDGF-BB (PDGF-B), PDGF-CC (PDGF-C), PDGF-DD (PDGF-D) and AB heterodimer (PDGF-AB)^7^. These interact in a specific manner with tyrosine kinase receptors of three different isoforms: PDGFR-αα (PDGFR-α), PDGFR-ββ (PDGFR-β) and αβ heterodimer (PDGFR- αβ). The different ligand isoforms have variable affinities for the receptor isoforms causing cross reactivity. PDGFR-β is activated by PDGF-B or PDGF-D.

Although several PDGFR inhibitors are approved for clinical use in other cancer types, attempts at PDGFR inhibition in PC patients have so far been unsuccessful with no improvement in disease specific survival, despite robust pre-clinical results^8^,^9^,^10^.

Alternations of PDGFRs have been detected in several cancers including pancreatic, ovarian, breast, gastric, thymoma, gastrointestinal stromal tumor, osteosarcoma, hepatocellular and hematologic cancers among others^11^,^12^,^13^,^14^,^15^. In PC, PDGF-D seems to be involved in osteoclastic differentiation and establishment of bone metastasis^16^. High levels of PDGFR-β in PC tumor stroma and non-malignant prostate tissue have been associated with shorter cancer specific survival for PC patients^17^. However, PDGFR-β expression for PC patients with a localized disease and its prognostic value post radical treatment has, to our knowledge, not been previously examined.

In our pursuit of new prognostic biomarkers and potential targets for novel therapeutic strategies, we systematically assessed both PC tumor and stromal expression of PDGFR-β and its ligands PDGF-B and PDGF-D, as well as associations with clinical outcome in a large multicenter cohort of 535 prostatectomy patients.

## Materials and Methods

### Patients

671 patients who underwent radical prostatectomy with curative intent for adenocarcinoma in the prostate from 1995 to 2005, were retrospectively identified from the Departments of Pathology at the University Hospital of Northern Norway (n = 267), Nordland Hospital (n = 63), St. Olavs Hospital (n = 330) and Levanger Hospital (n = 11). Of these, 136 patients were excluded due to (i) previous non-superficial cancer within five years of PC diagnosis (n = 4), (ii) radiotherapy to the pelvis prior to surgery (n = 1), (iii) inadequate paraffin-embedded tissue blocks (n = 130), and (iv) lack of follow-up data (n = 1), leaving a total of 535 eligible patients for the cohort. None of the patients had received pre-operative hormonal therapy. The cohort is thoroughly described in a previous paper^18^.

We collected relevant data from medical journals involving: Demographical data, age at surgery, previous medical history, retropubic or perineal surgery, and preoperative serum PSA level measured immediately before surgery. Further, we collected outcome data until the last follow-up date (December 01, 2015) or until patients’ death. The surviving patients’ disease-specific outcomes were recorded for a median follow-up of 12.4 years (range 1.5–20 years). These data included postoperative PSA values and postoperative therapy (radio-, hormonal- and/or chemotherapy). The following endpoints were used: Biochemical failure (BF) defined as postoperative PSA ≥ 0.4 or intervention with salvage therapy; Clinical failure (CF) defined as clinically palpable tumor recurrence in the prostate bed or metastasis verified by radiology; Prostate cancer specific death (PCD), defined as death caused by PC stated in the patients’ journal.

### Tissues and tissue microarray construction

Tumor tissues, consisting of formalin-fixed paraffin-embedded blocks of prostate tissue from the patients’ prostatectomies, were collected from the archives of the pathological departments. One experienced pathologist (E.R.) reevaluated the prostate samples and classified them according to the updated WHO guidelines^19^,^20^. Two pathologists (E.R. and L.T.B.) identified the most representative areas of cancer epithelium cells and adjacent stroma. Each area was biopsied with at least two 0.6 mm cores. The cores were arranged in tissue microarray (TMA) blocks for large-scale analysis. Multiple 4 μm TMA sections were cut with a Micron microtone (HM355S) and stained by specific antibodies for immunohistochemical analysis (IHC). The detailed methodology has been reported previously^21^.

### Immunohistochemistry

Immunohistochemical analysis was performed on Discovery-Ultra immunostainer (Ventana Medical Systems, Tucson, AZ). Slides were deparaffinized in three 8-minute cycles. On-board CC1 antigen retrieval incubated for PDGF-D, PDGF-B and PDGFR-β, 32, 24 and 48 minutes respectively. Discovery inhibitor (Cat #760–4840) blocked endogenous peroxidase for 8 minutes. The following primary antibodies were loaded: PDGF-D (R&D system, #AF1159, goat, polyclonal, 1/40 dilution), PDGF-B (Sigma, #A81363, rabbit, polyclonal, 1/25 dilution) and PDGFR-β (Cell Signaling, #3169, rabbit, monoclonal, 1/25 dilution). The slides were incubated for 32 minutes at 37 °C. Antibody dilution buffer (Ventana, #ADB250) were used for all antibodies except for PDGF-D where Discovery antibody diluent (Ventana, #760–108) was utilized. Slides were developed using corresponding secondary antibody for 20 minutes, followed by 12 minutes HRP amplification for PDGFR-β and were detected using ChromoMap DAB (Cat #760–159). Finally, the slides were counterstained to detect the nuclei with Ventana Hematoxylin II reagent for 32 minutes, followed by a Bluing reagent for 8 minutes and dehydrated, cleared and mounted as in our routine processing.

Two different controls for our staining method were applied. Firstly, control staining of the sections with an isotype-matched control antibody without the primary antibody. Secondly, multiple human organ TMA as positive and negative tissue controls were used to verify the specificity of the staining in every staining procedure. Positive tissue controls comprised of colon carcinoma and placenta for PDGFs, while negative tissue controls comprised of normal tonsil and brain.

### Scoring of immunohistochemistry

One experienced pathologist (E.R) and one experienced oncologist trained in assessing histopathological slides (S.A) independently and semiquantatively scored viable parts of each anonymized core by light microscopy. The scorers were blinded for each other’s score. Each core was scored by the dominant intensity of staining: 0 = no staining; 1 = weak staining; 2 = moderate staining; 3 = strong staining. In addition, each core was also scored by density according to the fraction of marker positive cells in stroma: 0 = 0% positive cells; 1 = 1–50% positive cells; 2 = 50–75% positive cells; 3 ≥ 75% positive cells. Stroma and epithelium were scored independently if the marker was expressed in these compartments. The core was scored as “missing” if the core was missing or considered of insufficient quality to score by both observers. A final score for both intensity and density marker expression in both epithelium and stroma for each patient was calculated using the mean values of the observers’ scoring of the patients cores. Scoring of IHC cores were dichotomized into low and high expressions. Cut-off values was set at median to secure reproducibility and statistically sufficient numbers in each group. High or low expression of PDGF-B or PDGF-D were not significantly associated with endpoints for any cut-off. For PDGFR-β, there was no expression of the marker in epithelium. In stroma though, there was a heterogeneous distribution of density (cut-off 1.50), while there was a relatively high expression of intensity (cut-off 2.25).

### Statistical methods

SPSS 23.0.0.0 (Chicago, IL) was used for all statistical analyses. Correlations were analyzed using Spearman’s rank correlation coefficient. Comparing means of expressions between different tissues were analyzed using the non-parametric Wilcoxon signed rank test. Univariate survival curves were drawn by the Kaplan-Meier method, and the statistical significant difference between survival curves was assessed by the log-rank test. Presentations of the survival curves were terminated at 194 months due to less than 10% of patients at risk after this point. For multivariate analyses, the backward conditional Cox-regression analysis was used with a probability for stepwise entry at 0.05 and stepwise removal of 0.10. A p < 0.05 was considered statistically significant for all analyses.

### Ethics

The reporting of clinicopathological variables, survival data and biomarker expressions was conducted in accordance with the REMARK guidelines. This study has been approved by The Regional Committee for Medical and Health Research Ethics, REK Nord, project application 2009/1393, including a mandatory reapprovement January 22, 2016. The committee waived the need for patient consent for this retrospective study. The Data Protection Official for Research (NSD) approved the establishment of the database.

## Results

### Clinicopathological variables and patient characteristics

The patients’ clinicopathological data are presented in the first part of Table 1. Median age at surgery was 62 (47–75) years. At the last follow-up, 37% of the patients had BF, 11% had CF and 3.4% were dead of PC. Median preoperative serum PSA was 8.8 (range 0.7–104) and the median tumor size was 20 mm (2.0–50).

### Expressions

For PDGF-D, intensity was scored in both tumor and normal epithelium. Stroma was not scored due to weak staining of fibromuscular stroma, and the positive staining in stroma was mainly in lymphoid cells. Density of PDGF-D was not scored due to homogenous distribution. While macrophages and lymphoid cells were positive stained, fibroblasts did not express PDGF-D. The staining was cytoplasmic and granular. PDGF-D was expressed at a higher level in tumor epithelium (mean = 2.13) compared to normal epithelium (mean = 1.85, p < 0.001).

For PDGF-B, only intensity was scored as density was homogenously distributed. Stroma could not be scored due to an overall strong staining of fibromuscular stroma. Intensity of both tumor epithelium and normal epithelium was scored separately in two groups. PDGF-B expression was overall cytoplasmic in the luminal and basal cells of the epithelium. There was no significant difference in PDGF-B expression in tumor epithelium (mean = 1.48) versus normal epithelium (mean = 1.52, p = 0.194).

Both intensity and density were scored for PDGFR-β. But since PDGFR-β was not expressed in epithelium, only stroma was scored. Both tumor stroma and normal stroma were scored into two separate groups. The staining was cytoplasmic and granular, and no membrane staining was seen. Intensity of PDGFR-β was higher in tumor stroma (mean = 2.35) compared to normal stroma (mean = 1.85, p < 0.001), and staining density was also higher in tumor stroma (mean = 1.85) compared to normal stroma (mean 1.28, p < 0.001).

**Figure 1:**
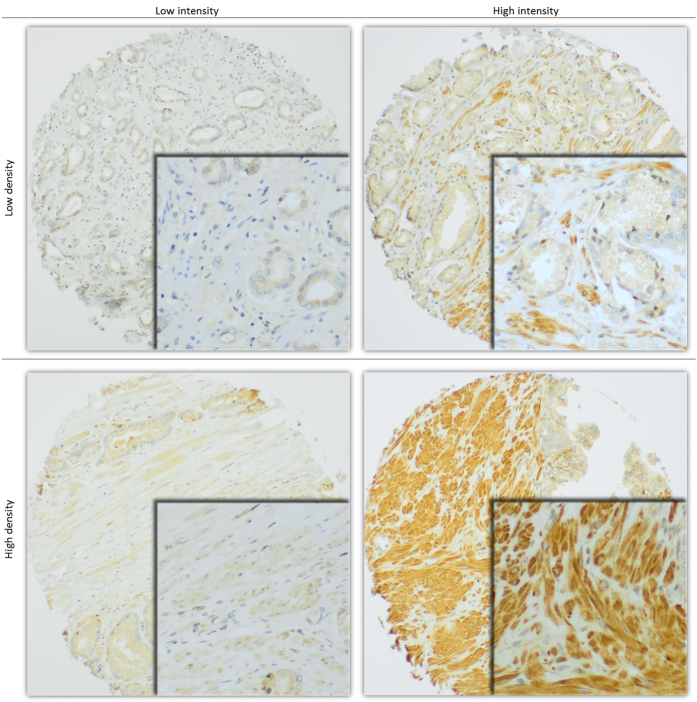
Examples of high and low intensity and density of PDGFR-β immunohistochemical staining in tissue microarray cores of prostate cancer stroma. 100× (main) and 400× (embedded) magnification.

Representative light microscopic examples of PDGFR-β high and low intensity and density are shown in Fig. 1.

### Correlations

There was a high intraclass correlation between the two scorers, with a correlation coefficient of 0.95 (CI = 0.94–0.95, p < 0.001). None of the biomarkers correlated to any of the clinicopathological variables except a weak correlation between mean density of PDGFR-β in stroma and perineural infiltration (r = 0.25, p < 0.001). For the cases where there were two valid scores of stroma or epithelium, the intra-case heterogeneity was calculated using the intraclass correlation procedure. For intensity of PDGFR-β stroma scores, there was a correlation coefficient of 0.78 (CI = 0.37–0.89, p < 0.001) of absolute agreement. For density of PDGFR-β stroma scores, the correlation coefficient was 0.79 (CI = 0.65–0.86, p < 0.001).

### Univariate analyses

Results from the univariate analyses of the clinicopathological variables are presented in Table 1. For BF, significant prognostic clinicopathological factors were pT-stage (p < 0.001), preoperative PSA (p < 0.001), Gleason score (p < 0.001), tumor size (p < 0.001), perineural infiltration (p < 0.001), lymphovascular infiltration (p < 0.001) and positive surgical margin (p = 0.049) with its subclass non-apical margin (p < 0.001). For CF, significant prognostic factors were age (p = 0.038), pT-stage (p < 0.001), preoperative PSA (p = 0.029), Gleason score (p < 0.001), tumor size (p < 0.002), perineural infiltration (p < 0.001), vascular infiltration (p < 0.001) and positive non-apical margin (p < 0.001). The significant prognostic factors for PCD (not presented in tables) were pT-stage (p < 0.001), preoperative PSA (p = 0.003), Gleason score (p < 0.001), perineural infiltration (p < 0.001), lymphovascular infiltration (p < 0.001) and positive non-apical surgical margin (p = 0.022).

Statistical analyses found no difference in endpoints with respect to expressions in tumor stroma respective normal stroma. Hence, all stromal scorings were pooled. Intensity and density of PDGFR-β in stroma versus endpoints in univariate analyses were examined. Results showed that both intensity and density of PDGFR-β were correlated to BF and CF, but density yielded stronger results in means of higher hazard ratio (HR) and significance than intensity. In addition, a backward Cox regression analysis, comparing intensity and density versus endpoints, was performed, and intensity was removed before the last step in the analysis. Hence, we chose to focus on expression as density of PDGFR-β in all stromal scorings.

**Figure 2:**
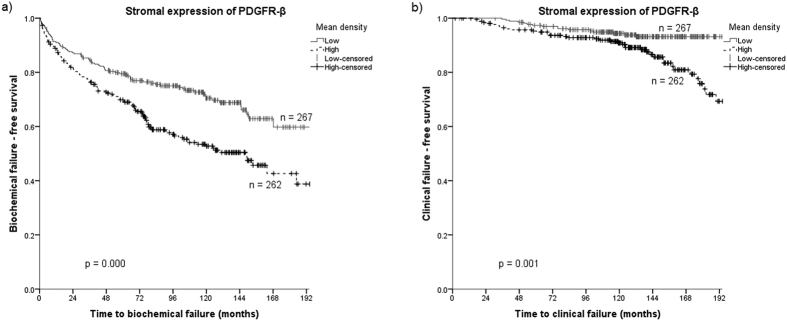
Kaplan-Meier curves of low and high expression of PDGFR-β in prostate cancer stroma for (**a**) biochemical failure and (**b**) clinical failure.

Results from the univariate analyses of the molecular markers according to BF and CF endpoints are presented in Table 1 and Fig. 2. Patients with a high expression of PDGFR-β in stroma had significantly worse outcome regarding BF (p < 0.001) and CF (p = 0.001) compared to patients with low expression of PDGFR-β. For PCD (3.4% of cases), no significant outcome difference was observed regarding high or low PDGFR-β expression subgroups.

Expression levels of PDGFR-β versus BF and CF stratified according to AJCC (American Joint Committee on Cancer) PC stage are presented in Table 2. For BF, high expression of PDGFR-β is associated with a worse outcome in stage IIB (p = 0.007) and III (p = 0.029). For CF, high expression of PDGFR-β is associated with a worse outcome in stage IIA (p = 0.011) and IIB (p = 0.027).

Univariate analyses of PDGF-B and PDGF-D expressions showed no significant associations with BF, CF and PCD.

### Multivariate analyses

Results from a multivariate model of clinicopathological variables and biomarkers are shown in Table 3. We observed that in addition to clinicopathological variables [pT-stage (p < 0.001), preoperative PSA (p = 0.014), Gleason 4 + 3 (p = 0.039), Gleason ≥ 9 (p = 0.018) and positive non-apical margin (p = 0.003)], a high expression of PDGFR-β in stroma correlates to a worse BF (HR = 1.58, p = 0.002). For CF, the only factors that correlate to a significantly worse outcome are Gleason score (p < 0.001) and high expression of PDGFR-β in stroma (HR 2.17, p = 0.010).

## Discussion

We demonstrate a high expression of PDGFR-β in prostate cancer stroma to be independently and significantly associated with biochemical and clinical recurrence in PC patients treated by radical prostatectomy. We found the mean expression of PDGFR-β to be higher in tumor stroma compared to normal stroma. In our cohort, PDGFR-β outperforms well-established prognostic factors like pT-stage, preoperative PSA, tumor size, PNI, lymphovascular infiltration and positive surgical margin as a prognostic tool. There was no significant difference in clinical outcome according to PDGF-B or PDGF-D expression.

PDGF pathway studies are scarce in PC and the majority has been performed *in vitro*. The absence of marker studies involving normal and malignant tissues in both epithelial and stromal compartments further underpins the need for further investigation in this field. The strengths of our study are the size of our multicenter cohort, the long clinical follow-up, and the examination of both tumor epithelium and stroma. In contrast to RT-PCR techniques, IHC markers allow us to visualize and assess the expression of antibodies *in situ*. Despite the long clinical follow-up (mean 12.4 years), the relatively low incidence of clinical recurrence and prostate cancer-specific death leaves a relatively low numbers of events. This demonstrates the need for even larger PC studies to properly evaluate these endpoints. This study is biased towards the selected group of patients that are considered healthy enough to undergo prostatectomy and towards stages of PC that are perceived as surgically curable.

In a phase II study of the PDGFR-inhibitor SU101 for patients with hormone-refractory PC, PDGFR-β was shown by IHC analysis to be upregulated in most primary and metastatic PC cells^22^. Corroborating our findings, Singh *et al*. revealed, by using a gene microarray on 235 tumor samples, that PDGFR-β is one of at least five genes that predict PC recurrence after prostatectomy^23^.

Hagglof *et al*. found that a high expression of PDGFR-β in both normal and tumor stroma was associated with poor survival and advanced disease in a natural course of the disease, without radical intervention^17^. Their study was based on PC tissue specimens collected from approximately 300 patients subjected to transurethral resection of the prostate (TURP) during 1975–1991. As radical treatment had not been implemented as medical practice at the time, their sampled TURP material differs from the intervention prostatectomies of our study. Our results show that high expression of PDGFR-β is a prognostic factor after prostatectomy intervention with curative intent, and as such unveils the importance of PDGFR-β expression at a more relevant clinical setting. While Hagglof *et al*. did not observe significant prognosticators in the multivariate analysis, our multivariate results showed that Gleason score and expression of PDGFR-β were independent significant prognostic factors for clinical failure. Although the clinical setting is different from the study by Hagglof *et al*., our results build on their results and demonstrate that PDGFR-β in either benign or malignant stroma of PC tissue is a prognostic biomarker both in the natural history of PC and after prostatectomy.

When investigating the ligands PDGF-B and PDGF-D, PDGF-D was expressed at a higher level in tumor epithelium compared to normal epithelium. Other studies have suggested that PDGF-D seems to be involved in development of bone metastasis, and is associated with increased Gleason and tumor stage^24^,^25^. However, we found no associations between expression of PDGF-D and clinical outcome. A reason for this may be that our sample selection consists of patients with localized disease, whereas earlier studies of PDGF-D have been studies implicating a more advanced disease^26^. Our results indicate that PDGF-D is not significantly associated with cancer relapse in earlier stages of the disease.

We found no difference in levels of PDGF-B expression in normal versus tumor epithelium, nor was there any associations between expressions and prognosis. These findings are supported by previous clinical studies demonstrating that both PDGFR-β and PDGF-D are up-regulated in primary prostate cancers and bone metastases, whereas PDGF-B is not frequently detected in clinical samples^27^. Hence, it is the upregulation of the receptor PDGFR-β that seems to be of clinical significance for patients considered for radical treatment.

In our cohort, the risk of BF increased 58% (HR 1.58) as a result of high PDGFR-β expression in stroma. Even more importantly, the only two factors that predict clinical failure in our cohort are Gleason score and high expression of PDGFR-β. If fact, high PDGFR-β expression more than doubles the risk of clinical failure. Expressions of PDGFR-β have a significant impact on BF and CF for the intermediate risk groups IIA, IIB and III. This is of particular interest as we are in desperate need for better prognostic tools in this patient group.

Our results show that both normal and malignant stroma are of clinical importance. The stromal microenvironment is an active and important biological compartment. Mediated through direct cell-cell contacts or by secreted molecules, there is a continuous and bilateral molecular crosstalk between both normal cells and tumor cells of the stromal compartment. Accordingly, minor changes in one compartment may cause dramatic alterations in the whole system^28^.

Treatment with inhibitors of the PDGF pathways has been established for several cancer types. The tyrosine kinase inhibitor (TKI) imatinib is a potent inhibitor of the PDGFR, and is used to treat gastrointestinal stromal tumors, some forms of leukemia, and myeloproliferative diseases among others. Despite robust pre-clinical data, imatinib has proven ineffective in Phase I and II clinical trials for patients with metastatic castration-resistant PC (mCRPC)^10^. A Phase II trial even showed that PDGFR inhibition with tandutinib was associated with accelerated disease progression, hypothesizing that PDGF contributes to the homeostasis of bone metastases from PC^8^. Other attempts of PDGF inhibition in PC has been no more successful^29^,^30^.

Although angiogenesis as endothelial sprouting is regarded as a hallmark of cancer development, several studies have shown primary tumors and metastases to be able to progress without angiogenesis^31^,^32^. The concept of vascular co-option implies that tumors can obtain blood supply by overtaking the native vasculature and let tumor cells migrate along the vessels of the host organ. Intussusception (or splitting angiogenesis) implies the mechanism where preexisting vessels split into daughter vessels. These relatively new considerations suggest that the vasculature of human tumors is more comprehensive than previously regarded, and have been introduced as a potential explanation of antiangiogenic drug resistance.

As a clinically and molecularly heterogeneous disease, the lack of available prognostic biomarkers for PC patient stratification regarding therapy is one of the key reasons why several trials have produced disappointing results. PDGFR upregulation has been suggested as a mechanism of evading different targeted drug therapies in some preclinical studies, and further exploration in a clinical relevant setting is warranted^33^. Specific prognostic biomarkers, associated with response to therapy, are also warranted in order to guide treatment stratification. There are still several unresolved aspects regarding PDGFR inhibition as PC treatment. Hitherto, no studies involving PDGFR-inhibition has been carried out in early stage prostate cancer. According to translational research data, it can be speculated that such therapy may prove effective in the primary setting.

In conclusion, our results indicate PDGFR-β in either benign or tumor associated stroma to be a strong, independent predictor of prostate cancer recurrence. Although PDGF inhibition so far has been disappointing, its implication in PC relapse warrants further exploration in an optimal setting. As a prognosticator, PDGFR-β in PC stroma consistently appears to be associated with poor prognosis, particularly in the important intermediate risk subgroups. Prospective validation should be considered for future studies.

## Additional Information

**How to cite this article:** Nordby, Y. *et al*. High expression of PDGFR-β in prostate cancer stroma is independently associated with clinical and biochemical prostate cancer recurrence. *Sci. Rep.* **7**, 43378; doi: 10.1038/srep43378 (2017).

**Publisher's note:** Springer Nature remains neutral with regard to jurisdictional claims in published maps and institutional affiliations.

## Figures and Tables

**Table 1:** Patient characteristics, clinicopathological variables and expressions of PDGFR-β in 535 prostate cancer patients (univariate analyses; log-rank test).

Characteristics	Patients		BF (200 events)		CF (56 events)	
	(n)	(%)	5 year EFS (%)	*p*	10 year EFS (%)	*p*
Age				0.237		**0.038**
≤65 years	357	67	77		94	
>65 years	178	33	70		91	
pT-stage				**<0.001**		**<0.001**
pT2	374	70	83		97	
pT3a	114	21	61		87	
pT3b	47	9	43		74	
Preop PSA				**<0.001**		**0.029**
PSA < 10	308	57	81		95	
PSA > 10	221	42	68		89	
Missing	6	1	—		—	
Gleason				**<0.001**		**<0.001**
3 + 3	183	34	83		98	
3 + 4	219	41	77		94	
4 + 3	81	15	70		90	
4 + 4	17	4	58		86	
≥9	35	6	37		65	
Tumor Size				**<0.001**		**0.002**
0–20 mm	250	47	83		96	
>20 mm	285	53	68		90	
Perineural infiltration				**<0.001**		**<0.001**
No	401	75	80		96	
Yes	134	25	60		83	
Lymphovascular infiltration				**<0.001**		**<0.001**
No	492	92	77		95	
Yes	43	8	47		69	
Positive surgical margin				**0.049**		0.198
No	249	47	81		96	
Yes	286	53	69		90	
Apical positive surgical margin				0.063		0.427
No	325	61	74		92	
Yes	210	39	77		93	
Non-apical positive surgical margin				**<0.001**		**<0.001**
No	381	71	82		96	
Yes	154	29	57		85	
Surgical procedure				0.466		0.308
Retropubic	435	81	77		92	
Perineal	100	19	68		95	
PDGFR-β in stroma				**<0.001**		**0.001**
Low expression	267	50	80		94	
High expression	262	49	70		91	
Missing	6	1				

Abbreviations: BF = biochemical failure; CF = clinical failure; EFS = event free survival in months.

**Table 2:** Ten year EFS for patients with low or high levels of PDGFR-β stromal expression in relation to prognostic groups of prostate cancer.

		10 year EFS (%)					
		Biochemical failure			Clinical failure		
Group		Low expr	High expr	p	Low expr	High expr	p
I	(n = 43)			NS			NS
IIA	(n = 111)	76	64	0.082	100	92	0.007
IIB	(n = 219)	82	64	0.007	98	96	0.026
III	(n = 159)	48	29	0.029			NS
IV	(n = 3)			NS			NS

The stratification of our cohort into prognostic groups are constructed according to the American Joint Committee on Cancer (AJCC) TNM system.

Abbreviations: EFS = Event free survival; NE = No events; NS = Not significant (p > 0.10); expr = expression of PDGFR-β.

**Table 3:** Expression of PDGFR-β in prostate tissue as a prognostic factor in 535 prostate cancer patients (multivariate analyses; Cox regression with backward conditional model).

Characteristics	BF (200 events)			CF (56 events)		
	HR	CI 95%	*p*	HR	CI 95%	*p*
Age	NE			NS		
pT-stage			**<0.001**	NS		
pT2	1					
pT3a	1.56	1.07–2.25	**0.019**			
pT3b	2.46	1.55–3.90	**<0.001**			
Preop PSA			**0.014**	NS		
PSA < 10	1					
PSA > 10	1.45	1.08–1.95				
Gleason			0.064			**<0.001**
3 + 3	1			1		
3 + 4	1.19	0.82–1.70	0.360	3.37	1.36–8.37	**0.009**
4 + 3	1.59	1.02–2.47	**0.039**	4.45	1.61–12.3	**0.004**
4 + 4	1.98	0.98–4.00	0.058	5.40	1.35–21.7	**0.017**
≥9	1.95	1.12–3.38	**0.018**	15.1	5.83–39.2	**<0.001**
Tumor Size	NS			NS		
0–20 mm						
>20 mm						
Perineural infiltration	NS			NS		
No						
Yes						
Lymphovascular infiltration	NS			NS		
No						
Yes						
Non-apical positive surgical margin			**0.005**	NS		
No	1					
Yes	1.57	1.15–2.15				
PDGFR-β in stroma			**0.002**			**0.010**
Low expression	1			1		
High expression	1.58	1.18–2.13		2.17	1.20–3.90	

Abbreviations: BF = biochemical failure; CF = clinical failure; NE = not entered into Cox regression due to not significant in univariate analyses; NS = not significant and removed by backward model before last step of analyses.
